# Incidence of non-affective psychotic disorders in refugees and peers growing up in Denmark and Sweden: a registry linkage study

**DOI:** 10.1007/s00127-023-02578-x

**Published:** 2023-11-02

**Authors:** Christopher J. de Montgomery, Alexis E. Cullen, Heidi Taipale, Allan Krasnik, Marie Norredam, Ellenor Mittendorfer-Rutz

**Affiliations:** 1 Department of Public Health, Danish Research Centre for Migration, Ethnicity and Health (MESU), University of Copenhagen, Øster Farimagsgade 5, 1014 Copenhagen, Denmark; 2 Department of Clinical Neuroscience, Division of Insurance Medicine, Karolinska Institutet, Stockholm, Sweden; 3 Department of Psychosis Studies, Institute of Psychiatry, Psychology & Neuroscience, King’s College London, London, UK; 4 Niuvanniemi Hospital, Kuopio, Finland; 5 School of Pharmacy, University of Eastern Finland, Kuopio, Finland; 6 Section of Immigrant Medicine, Department of Infectious Diseases, University Hospital Hvidovre, Copenhagen, Denmark; 7 Section for Science of Complex Systems, CeMSIIS, Medical University of Vienna, Vienna, Austria

**Keywords:** Comparative study, Incidence, Non-affective psychotic disorders, Refugees, Migrants

## Abstract

**Purpose:**

Higher rates of non-affective psychotic disorders (NAPD) in minority groups have been reported in many countries. However, few studies have explored how rates differ between refugees and other minority groups and none with an international comparative angle. A comparative perspective makes it possible to relate group differences to aspects national context that underpin the social determinants of disease.

**Methods:**

We compared the incidence of treated NAPD among youth born in or who immigrated to Denmark/Sweden before turning 18. Youth aged 18–35 during 2006–2018 were included (N_Denmark_/N_Sweden_ = 1,606,423/2,614,721) and were followed until first NAPD treatment (cases [Denmark/Sweden] = 12,193/9,641), 36^th^ birthday, emigration or death. Incidence rates (IR) and ratios (IRR) comparing refugees, non-refugee migrants, descendants of non-refugee migrants and majority youth were obtained through Poisson regression on data aggregated by country, sex and age, contrasted by sex and country. Complementary analyses on individual-level data adjusting for further socio-demographic factors were conducted in each country separately.

**Results:**

Incidence rates were higher in all groups compared with the majority group (IRR_range_ = 1.4–2.9, 95% CI_[min, max]_ = 1.2–3.1). Relative differences between the three minority groups were smaller (IRR_range_ = 0.7–1.0, 95% CI_[min, max]_ = 0.5–1.2). Although incidence rates were higher in Denmark than Sweden, relative group differences were similar.

**Conclusion:**

Exposures shared between young refugees and other minority groups growing up in Denmark and Sweden may be especially important for their excess risk of NAPD. Further studies should investigate the mechanisms behind the elevated rates in minority groups with special paid attention to factors such as discrimination, social exclusion and acculturation stress.

**Supplementary Information:**

The online version contains supplementary material available at 10.1007/s00127-023-02578-x.

## Introduction

Non-affective psychotic disorders (NAPD) have serious consequences in terms of both increased mortality [1] and loss of quality of life and standard of living [2]. With a typical onset during late adolescence and early adulthood, individuals are often challenged to complete education and training and to become self-providing [3, 4]. Tackling inequities that give rise to elevated rates and worse prognosis in certain population groups is a critical public health issue.

Elevated rates of NAPD have been reported in young refugees resettled in several high-income countries [5–7] and in other groups of migrants and descendants of migrants [8–10]. Childhood trauma has been shown to be associated with psychosis in both retrospective and prospective studies [11, 12] and is particularly prevalent among youth who have been forcibly displaced [13, 14]. But exposure to other risk factors in the aetiology of psychosis including experiences of discrimination, socio-economic adversity, social isolation and distress [15, 16] are more widespread across groups of migrants and minorities. In addition, these factors are amenable to action within the countries of residence, which underscores the importance of direct comparisons of rates between groups across country contexts.

The relatively low incidence of psychotic disorders combined with the moderate sizes of refugee populations within many high-income countries makes it challenging to estimate the extent to which the incidence differs among refugees and other non-majority groups. Evidence from Sweden does show that refugees are at higher risk of NAPD than other migrant groups [7], but not higher than adoptees from the same geographical regions [17]. No previous study has made direct comparisons between refugees and other minority groups in an international comparative design.

The comparison of Denmark and Sweden offers a unique context in which to explore how incidence of NAPD and age-patterns in healthcare contact vary across population groups and healthcare systems. Following early pioneering work in the UK and Australia [18], Denmark was swift to join the early intervention movement for psychosis among young people and has contributed to the international evidence base behind it with large randomized trials [19–23]. For a decade and a half, Denmark has had national guidelines to implement early intervention services for psychosis in all healthcare regions [24, 25]. These early intervention teams combine assertive community treatment, sessions for family members, and social skills training, bolstered by resources that permit a staff: patient ratio 2–3 times smaller than standard treatment [25]. In contrast, Sweden has not had a national focus on early intervention although isolated trials have been attempted in some parts of the country [26]. This difference may have implications for both the timing of diagnosis and the diagnostic practice for youth presenting with psychotic symptoms. While the general immigration trends are similar in Denmark and Sweden, Sweden has since the 1980s received a much larger number of refugees and the proportion of immigrants and descendants of immigrants is almost twice as high in Sweden than Denmark [27, 28]. Sweden has also placed greater emphasis on adapting services to its more diverse population as in Denmark [29], for example by providing care for asylum seekers within the national health system rather than through parallel services [30], which may have consequences both for exposures to stressors related to NAPD and for inequalities in accessing healthcare. Finally, in both countries high-quality registry data makes it possible to conduct studies with full national populations that have the necessary sample sizes to investigate psychotic disorders in narrowly defined groups.

The objectives of this study were to compare the incidence rates of NAPD and age at first treatment in young refugees, non-refugee migrants, descendants of non-refugee migrants and majority youth in two different national contexts in a comparative design. As incidence rates and age-patterns differ between women and men [31], analyses were stratified by sex. In addition, we investigated the role of factors associated with socio-economic marginalization in each country separately. We hypothesized that group differences would be smaller in Sweden than in Denmark, as Sweden is a more diverse society with more migrant friendly policies, while NAPD patients would be younger at first treatment in Denmark given the availability of early intervention services in Denmark.

## Methods

The study utilized nationwide registers from Denmark and Sweden. Psychiatric care data stemmed from records from secondary care, including in- and specialized out-patient as well as emergency contacts, including information on discharge diagnosis and timing of admission and discharge or of outpatient contact. Information on purchases of prescribed antipsychotic medication [Anatomic Therapeutic Chemical classification (ATC) codes N05A, omitting lithium N05AN] was available in both countries, but did not include medication dispensed within inpatient settings. Demographic and socio-economic data was available in both countries. All registers were linked through the anonymized unique personal identification code.

As individual-level data could not be transferred across countries, our comparative analyses relied on pooled aggregated data stratified by group, age-group (three-year bands), sex and country. Complementary analyses with individual-level data including further covariates were conducted separately in each country.

### Study populations

In each country, we coded four groups: refugees, non-refugee migrants, descendants of non-refugee migrants, and majority peers. The refugee group consisted of all immigrants to Denmark/Sweden who immigrated during 1986–2014 whose grounds for residence was registered with immigration authorities as refugee status or family reunification with a refugee. Only individuals aged 17 or younger at the time they obtained residency were included as we wanted to compare age-specific incidence among those living in the country during the follow-up ages from 18 to 35. In Denmark, grounds of residence data were only available from 1993, so for immigrants during 1986–1992 refugee status was determined by originating from one of the major refugee-sending countries during this period (Afghanistan, Lebanon (primarily stateless Palestinians), Iran, Iraq, Somalia, Sri Lanka and Vietnam). The (first-generation) migrant group comprised other immigrants with the same criteria as for the refugee group, except the grounds of residence for this group was not refugee status. While diverse in origins, this group shares with refugees the experience of migration during childhood, albeit not forced. The descendant group (second-generation) was born in Denmark/Sweden to immigrant parents (i.e. neither parent was born in Denmark/Sweden) and whose mother (or father if information on mother was missing) was born in (or a citizen of, if place of birth was unknown) Pakistan, Morocco or Turkey. Although findings related to this group of descendants may not be generalizable to descendants whose parents immigrated from other countries, this restrictive definition provides some context for the interpretation of the rates absent of information on grounds of residence for their parents’ generation. These minority communities were substantial in size in both countries and had a similar migration history, primarily the descendants of labour migrants from the 1960 and 1970s and their reunited family members. Like migrants and refugees, this group was likely to grow up as members of visible minorities that can involve common experiences, such as acculturation stress and discrimination, but they differed in that they were born and raised in Denmark/Sweden. Finally, the majority group included individuals born in Denmark/Sweden with a least one parent who was also born in Denmark/Sweden. While children of mixed heritage could be classified as both descendant and majority, we chose to include them in the much larger majority group to minimize the possible attenuation bias caused by misclassification.

### Follow-up and washout

All residents in Denmark/Sweden aged 18–35 (born 1971–1998), who had lived in the country for three full calendar years and who had no psychosis-related contacts during this period, entered the study on 1st of January 2006 or on their 18th birthday during the follow-up period from 2006 to 2018. As only year of birth was available in our dataset in Sweden, date of birth was defined as 1st of January in both countries. Three full calendar years of residence prior to entering the study was required to harmonize the washout period across populations, including refugee migrants, to distinguish incident from prevalent cases. Additionally, cases with records of purchased antipsychotic medication during the period 3–15 months prior to their first psychosis-related hospital contact were excluded, as such purchases would indicate an on-going treatment (possibly initiated in the primary healthcare sector or before study entrance). Purchases of antipsychotic medication during the three months leading up to their first contact were defined as part of the healthcare pathway related to first contact. Although medication dispensed in inpatient settings were not included in the data material, it is unlikely that individuals obtaining antipsychotic medication at hospitals would not be identified through the patient registry.

Individuals were followed from cohort entry until (i) date of first secondary care record (in- or out-patient) with a main discharge diagnosis in the F20-F29 range (NAPD) according to the 10th International Classification of Diseases (ICD-10), (ii) date of death, (iii) the 31st of December in the last calendar year that they were registered as residents in Denmark/Sweden, (iv) their 36th birthday, or (v) the end of the study period on 31st of December 2018.

### Covariates

For the individual-level analyses, it was possible to include further covariates. Living in a low-income household at age 18 (the youngest age at which data was available for population groups) was used as an indicator of material deprivation during adolescence. Household incomes were equivalized using the square root method [32] to account for household sizes, and a low income household was defined as an annual income below 60 pct. of the median that year. Municipalities were categorized as cities or other using EUROSTAT’s degree of urbanization (DEGURBA) classification of local administrative units [33], and measured as the domicile municipality at age 18.

Experiences of unemployment and disability pension (defined as any registered days during each year) and sickness absence (defined as any registered period longer than 30 days) were measured with a one-year time-lag, i.e. coded yearly during follow-up the year prior to case-ascertainment.

### Analytical procedures

For the main analysis, we estimated a Poisson model on the pooled data with person-time as an offset. First, a saturated model was estimated that included all interactions between group, age-group, sex and country. We then simplified the model stepwise using likelihood ratio tests. As interpretation of the model parameters was complicated by multiple interactions, we calculated marginal predictions and linear contrasts to present incidence rates (IR) and incidence rate ratios (IRR). A full regression results table is available in the supplementary materials (Table A1), as well as age-specific incidence (with 95% CI) in each group, for men and women in Denmark and Sweden (Table A2).

**Table 1:** Characteristics of the study populations in Denmark and Sweden

	Denmark								Sweden							
	Refugees		Migrant		Descendants		Majority		Refugees		Migrant		Descendant		Majority	
	Men	Women	Men	Women	Men	Women	Men	Women	Men	Women	Men	Women	Men	Women	Men	Women
Total populations (*n*)	18,776	15,536	15,894	16,185	14,072	13,705	760,262	725,088	84,423	68,721	64,027	65,131	12,372	11,664	1,253,036	1,184,543
Psychosis cases (*n*)	483	179	313	162	259	118	6229	4450	947	363	552	309	111	46	4739	2574
F2 diagnosis at first contact																
Schizophrenia (F20) (%)	32.7	31.5	33.0	25.5	30.1	35.0	34.8	33.7	9.3	6.1	9.1	5.5	6.3	4.4	6.4	5.4
Schizotypal (F21) (%)	7.2	8.4	12.2	14.9	6.6	16.2	24.8	24.8	0.2	0.3	0.9	1.3	0.0	2.2	1.9	1.3
Delusional (F22) (%)	12.6	14.6	13.1	8.1	16.2	6.0	9.0	6.7	7.1	7.4	6.9	11.3	11.7	8.7	8.6	8.1
Brief psy. epi. (F23) (%)	32.9	34.8	31.4	35.4	35.5	27.4	20.4	20.2	33.2	37.7	29.7	39.8	17.1	30.4	36.3	42.0
Other (F24–29) (%)	14.5	10.7	10.3	16.1	11.6	15.4	10.9	14.6	50.3	48.8	53.4	42.1	64.9	54.4	46.8	43.2
Type of admission																
In-patient (%)	38.3	37.6	43.9	44.7	37.8	29.9	30.0	27.7	55.9	57.6	54.4	56.6	49.6	41.3	51.8	51.2
Spec. out-patient (%)	61.7	62.4	56.1	55.3	62.2	70.1	70.0	72.3	44.1	42.4	45.6	43.4	50.4	58.7	48.2	48.8
Low-inc family^a^ (%)	31.7	30.0	15.0	15.7	18.6	19.1	7.4	8.8	32.8	28.1	23.1	24.0	17.5	18.4	6.1	8.5
Urban city^a^ (%)	37.3	38.4	31.3	31.6	63.8	64.1	23.8	24.0	46.3	49.0	45.9	45.0	68.4	68.4	28.0	28.2
Socio-econ. covariates^b^																
Unemployment (%)	51.8	53.3	19.2	24.2	42.4	47.3	36.6	45.1	59.5	55.2	47.0	43.2	51.5	47.9	41.0	39.8
Sickness absence (%)	15.6	15.5	4.8	6.6	14.4	16.7	12.9	17.8	12.8	22.3	10.9	21.3	14.9	28.6	12.9	24.9
Disability pension (%)	2.8	2.5	0.9	0.7	2.2	1.9	2.0	1.9	2.8	2.7	4.4	4.7	3.6	3.3	3.4	3.8
Age at first contact median	25	25	25	24	24	23	24	22	24	25	25	26	25	26	25	26
(IQR)	(22–28)	(21–29)	(21–29)	(21–28)	(21–28)	(21–28)	(21–28)	(20–27)	(21–29)	(21–29)	(22–30)	(22–31)	(22–30)	(23–30)	(22–29)	(22–30)

^a^Measured year turning 18

^b^At any point during study period

**Table 2:** Age-standardized incidence rates (IR) and incidence rate ratios (IRR) with 95% confidence intervals (CI) of non-affective psychotic disorders among refugees, migrants, descendants and majority youth

	Denmark				Sweden				Country dif.^d^ IRR [95% CI]
	Cases (py^a^)	IR^b^ [95% CI]	IRR^c^ [95% CI]		Cases (1000 PYs)	IR^b^ [95% CI]	IRR^c^ [95% CI]		
Men									
Refugees	483	285	2.8	Ref	947	140	2.9	Ref	0.5
	(166)	[259–311]	[2.5–3.1]	–	(652)	[131–149]	[2.7–3.1]	–	[0.4–0.5]
Migrants	313	248	2.4	0.9	859	97	1.9	0.7	0.4
	(122)	[220–276]	[2.1–2.7]	[0.7–1.0]	(989)	[90–103]	[1.8–2.1]	[0.6–0.7]	[0.3–0.4]
Descendants	259	226	2.2	0.8	111	108	2.2	0.8	0.5
	(115)	[197–255]	[1.9–2.5]	[0.7–0.9]	(104)	[87–128]	[1.8–2.7]	[0.6–0.9]	[0.4–0.6]
Majority	6229	107	Ref	0.4	4739	49	Ref	0.4	0.5
	(5791)	[104–110]	–	[0.3–0.4]	(9658)	[47–50]	–	[0.3–0.4]	[0.5–0.5]
Women									
Refugees	179	126	1.8	Ref	363	65	2.3	Ref	0.5
	(136)	[108–145]	[1.5–2.1]	–	(553)	[58–72]	[2.1–2.6]	–	[0.4–0.6]
Migrants	162	127	1.7	1.0	647	60	2.1	0.9	0.5
	(122)	[107–147]	[1.5–2.1]	[0.8–1.2]	(1138)	[55–65]	[1.9–2.3]	[0.8–1.0]	[0.4–0.6]
Descendants	118	101	1.4	0.8	46	49	1.7	0.7	0.5
	(110)	[82–120]	[1.2–1.7]	[0.6–1.0]	(97)	[35–64]	[1.3–2.3]	[0.5–1.0]	[0.3–0.7]
Majority	4450	80	Ref	0.6	2574	28	Ref	0.4	0.4
	(5524)	[78–83]	–	[0.5–0.7]	(9115)	[27–29]	–	[0.4–0.5]	[0.4–0.4]

Poisson model with group, sex, age (3-year categories) and country (pooled aggregated data)

^a^1000 person-years (rounded)

^b^Incidence rate per 100,000 person-years, post-estimation linear predictions based on model including group, sex, age and country and interactions between these (see supplementary Table A1 for full overview of interactions)

^c^Post-estimation linear contrasts

^d^Post-estimation linear contrast with Sweden as the reference group

Complementary analyses of overall group differences adjusted for further covariates were conducted with individual-level data separately in Denmark and Sweden (stratified for sex). Age, unemployment, disability pension, and sickness absence were included as time-varying covariates. Incidence rate ratios were estimated using Poisson regression with clustered standard errors to account for repeated observations of individuals, adjusting for covariates. Analyses were conducted in Stata/IC 15 and 16 [34, 35].

### Supplementary analysis

The one-year prevalence, i.e. the proportion of the full population who had a psychiatric healthcare contact each calendar year, was estimated for NAPD and other disorders during 2003–2018. The estimates are reported for 20-year olds and 50-year olds in Denmark and Sweden in the supplementary materials (Figure A1 and A2). These plots illustrate how psychiatric healthcare usage evolved in the two countries during the study period, which provides context for the interpretation of the rates in focus. We further reproduced the main analyses without exclusion of cases with prior antipsychotic medication claims.

## Results

### Sample characteristics

The study included a combined 21,834 cases of NAPD among 4,323,435 individuals in Denmark and Sweden (Table 1). While schizophrenia (ICD-10 code F20) accounted for 26–35% of cases across groups in Denmark, it only accounted for 4–9% of cases in Sweden. The bulk of first NAPD diagnoses given in Sweden were either brief psychotic episode (F23) or in the F24–F29 range within which 90–100% of diagnoses in Sweden were in fact ‘unspecified nonorganic psychosis’ (F29) (not shown). Only 10–16% of first NAPD diagnoses in Denmark were in the F24–29 range. Conversely, schizotypal disorders (F21) accounted for 25% of cases in the majority group in Denmark, but only 1–2% in Sweden. In terms of mode of access, specialized outpatient care was more commonly the site for the first registration of an NAPD diagnosis in Denmark than in Sweden.

Living in a low-income household was most common in the refugee groups in both Denmark and Sweden (28.1–32.8%), and least common in the majority groups (6.1–8.8%) (Table 1). In both countries, living in an urban municipality was most common in the descendant groups (63.8–68.4%) and least common in the majority groups (23.8–28.2%). For unemployment, sickness absence and disability pension (registered at any point during follow-up), the non-refugee migrant group in Denmark stood out with much lower rates than the other groups in both countries. While unemployment was most common among refugees in both countries (51.8–59.3%), levels of sickness absence were similar for men across groups in both countries (12.8–15.6%) and for women in Denmark (15.5–17.8%), but higher for women in all groups in Sweden (21.3–28.6%). Disability pension was rare in both countries (0.7–4.7%), but in Denmark highest for refugees (2.5–2.8%) and lowest for non-refugee migrants (0.7–0.9%), while in Sweden lowest for refugees (2.7–2.8) and highest for non-refugee migrants (4.4–4.7%).

### Incidence rates and ratios

Age-adjusted incidence ratios were almost identical for refugee men in Denmark (IRR = 2.8, 95% CI = 2.5–3.1) and Sweden (IRR = 2.9, 95% CI = 2.7–3.1) compared with the majority population (Table 2). This was also the case for men in the descendant group (IRR_Denmark_ = 2.2, 95% CI = 1.9–2.5; IRR_Sweden_ = 2.2, 95% CI = 1.8–2.7), while the ratios were slightly higher for migrants in Denmark (IRR = 2.4, 95% CI = 2.1–2.7) than Sweden (IRR = 1.9, 95% CI = 1.8–2.1). Among women, group differences were a little smaller in Denmark (IRR_refugees_ = 1.8, 95% CI = 1.5–2.1; IRR_migrants_ = 1.7, 95% CI = 1.5–2.1; IRR_descendants_ = 1.4, 95% CI = 1.2–1.7) than in Sweden (IRR_refugees_ = 2.3, 95% CI = 2.1–2.6; IRR_migrants_ = 2.1, 95% CI = 1.9–2.3; IRR_descendants_ = 1.7, 95% CI = 1.3–2.3). The smaller relative differences among women in Denmark seemed to depend on a higher incidence rate among majority women in Denmark, as rates were 38–56% lower among women than men in all other groups in both countries, but only 25% lower for majority women in Denmark.

While incidence rates were highest among refugees in both Denmark and Sweden, among both women and men, the relative differences between refugees, migrants and descendants were smaller and only significantly different among male descendants in Denmark (IRR = 0.8, 95% CI = 0.7–0.9) and male immigrants (IRR = 0.7, 95% CI = 0.6–0.7) and descendants (IRR = 0.8, 95% CI = 0.6–0.9) in Sweden. In Sweden, the incidence rates were half the magnitude or less compared with Denmark across groups (IRR_range_ = 0.4 to 0.5, 95% CI_max min_ = 0.3 to 0.7).

**Fig. 1:**
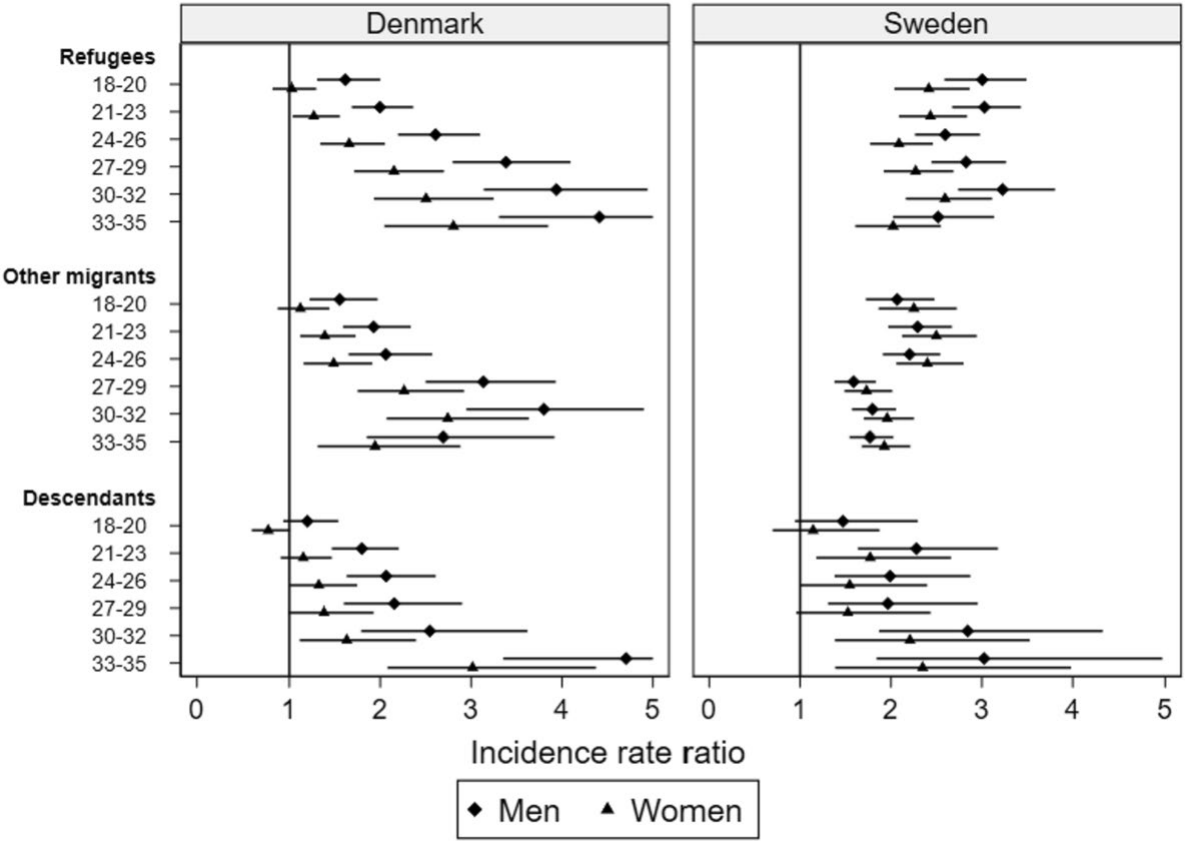
Incidence rate ratios (IRR) of non-affective psychotic disorders in Denmark and Sweden. Poisson model on aggregated data. Post-estimation linear contrasts with majority population as the reference group within each age/sex stratum. The reference line indicates no difference to the reference group, while point estimates are marked with 95% confidence intervals (capped at 5)

The relative differences between the majority group and the three comparison groups increased with age in Denmark, but not in Sweden (Fig. 1). In Denmark, this trend was driven by a sharp decline in incidence rates over age in the majority group combined with a smaller decline or no decline in the other groups (Table A2).

In the complementary analyses on individual-level records, we found that urbanicity, low income, unemployment, sickness absence and disability pension were associated with higher rates of NAPD in both countries (Table 3). However, while adjusting for these risk factors attenuated the relative differences between groups, the general trends remained the same. The adjusted ratios for descendant women in Denmark were an exception to this rule, as the adjusted rates were not significantly different from rates in the majority group at the 5% level.

**Table 3:** Incidence rate ratios and 95% confidence intervals showing population differences in non-affective psychotic disorders (ICD-10 codes F20–F29) among refugees, migrants and descendants compared with majority youth adjusted for socio-demographic risk factors

	Simple model		Full model	
	Men IRR [95% CI]	Women IRR [95% CI]	Men IRR [95% CI]	Women IRR [95% CI]
**Denmark (person-years)**	(6,180,192)	(5,870,651)	(6,180,192)	(5,870,651)
Population (ref: majority)				
Refugees	2.54***	1.55***	1.96***	1.21*
	[2.32–2.79]	[1.33–1.80]	[1.78–2.16]	[1.04–1.41]
Migrants	2.26***	1.55***	1.75***	1.19*
	[2.01–2.53]	[1.33–1.82]	[1.55–1.97]	[1.01–1.40]
Descendants	1.90***	1.13	1.51***	0.90
	[1.68–2.15]	[0.94–1.36]	[1.33–1.72]	[0.75–1.09]
Socio-economic variables				
Disability pension^a^			2.42***	2.56***
			[2.13–2.75]	[2.16–3.04]
Unemployment^a^			1.58***	1.23***
			[1.48–1.68]	[1.13–1.34]
Sickness absence^a^			2.33***	2.80***
			[2.12–2.57]	[2.50–3.12]
Urban municipality^b^			1.31***	1.32***
			[1.24–1.37]	[1.24–1.40]
Low income family^b^			2.42***	2.56***
			[2.13–2.75]	[2.16–3.04]
**Sweden (person-years)**	(10,854,560)	(10,205,232)	(10,854,560)	(10,205,232)
Population (ref: majority)				
Refugees	2.85***	2.30***	2.19***	2.05***
	[2.65–3.05]	[2.06–2.57]	[2.03–2.36]	[1.83–2.30]
Migrants	2.57***	2.50***	2.04***	2.16***
	[2.35–2.81]	[2.22–2.81]	[1.86–2.23]	[1.91–2.44]
Descendants	2.11***	1.67***	1.74***	1.48**
	[1.75–2.55]	[1.24–2.23]	[1.44–2.10]	[1.10–1.98]
Socio-economic variables				
Disability pension^a^			5.37***	5.84***
			[4.96–5.82]	[5.26–6.48]
Unemployment^a^			2.69***	2.06***
			[2.55–2.84]	[1.90–2.24]
Sickness absence^a^			2.74***	2.12***
			[2.49–3.01]	[1.90–2.37]
Urban municipality^b^			1.25***	1.14***
			[1.19–1.32]	[1.06–1.23]
Low income family^b^			1.61***	1.38***
			[1.50–1.73]	[1.26–1.53]

Poisson model (individual-level data, replication across countries)

Age and age squared included as continuous terms and year of entry included as fixed effects in all models

^a^Time-varying covariate measured preceding year

^b^Measured at age 18

*p < 0.05, **p < 0.01, ***p < 0.001

### Supplementary analysis

The one-year prevalence for any psychiatric disorder increased steadily from 2003–2018 among 20-year olds in both Denmark and Sweden (Figure A1). However, for NAPD a similar increase was observed only in Denmark. For 50-year-olds, the one-year prevalence rates were largely stable in both countries, except for the period 2003–2009 in Sweden (Figure A2), during which period data coverage of outpatient care improved notably in Sweden. While the one-year prevalence rates for NAPD were consistently higher in Denmark than Sweden during the whole period among 20-year olds, the rates were consistently higher in Sweden among 50-year olds. Finally, we reproduced the main analysis without excluding patients with prior antipsychotic medication claims (not shown). This yielded higher incidence rates in both Denmark and Sweden, but did not affect group or country differences.

## Discussion

This study is the first to conduct a direct comparison of differences in incidence rates of NAPD between young refugees, non-refugee migrants, descendants of non-refugee migrants, and majority youth across two countries. Our work yields two main findings. First, the difference in rates between the majority group and the non-majority groups were larger than between refugees, non-refugee migrants and descendants of migrants. Although we found that disability pension, sickness absence, experiences of unemployment and living in a low-income household were associated with higher rates of NAPD in all groups in both countries, these group differences persisted after adjustment for these factors. Second, the incidence rates and age patterns for first contact differed notably in Denmark and Sweden. Across groups, rates were 2–3 times higher in Denmark than in Sweden and the timing of first contacts was more heavily concentrated around the ages 18–24 in Denmark in the majority group in particular. In consequence, group differences increased with age in Denmark.

In terms of magnitude, incidence rate ratios for refugees compared with the majority population were similar to the results of the meta analysis by Brandt et al. [5], but higher for non-refugee migrants. This could be due to our restrictive approach to distinguish incident cases from prevalent cases, as well as the sample restriction that excluded individuals who immigrated after age 18, which effectively meant that we excluded more transient migrant communities and focused the analysis a population that spent at least a portion of their childhood in Denmark or Sweden. However, the IRR for immigrants and descendants were similar to those reported in international reviews and meta-analyses [36, 37], which also have not found significant differences between first and second generation immigrants. While we compared a heterogeneous non-refugee migrant group with descendants from particular origin countries, it is noteworthy that the absence of significant differences between the first- and second-generations appears robust to such choices of classification.

These trends point towards the importance of risk factors that play out within the countries of residence and affect both new migrants and locally born ethnic minorities, such as socio-economic adversities [11] and experiences of discrimination [38]. Interestingly, studies from both Denmark/Sweden and other countries have identified a protective role for living in communities with others from the same ethnic background [39–41], especially strong for probable visible minorities [39], which may serve to counter feelings of social isolation and serve as a buffer against experiences of discrimination [16]. As group differences in rates were similar in Denmark and Sweden, these factors may affect minorities similarly in the two countries despite the more immigrant-friendly policy stands in Sweden during this period [42].

However, the country-level differences in rates and timing of first contact in Denmark and Sweden are notable in the light of the fact that Denmark has an established system for early intervention targeting youth with first-episode psychosis, while Sweden does not. This system was further expanded and consolidated during the study period from 2006 to 2018 [25]. The higher rates and the younger age at first contact in Denmark, as well as greater propensity to make contact in outpatient settings, could suggest that such services do bring youth with first-episode psychosis into treatment sooner, and testing this hypothesis could be an avenue for future research. One reason why group differences increased with age in Denmark could be that non-majority groups experience longer delays before entering treatment after the onset of symptoms. If the increasing group differences at higher ages were explained by a later onset of psychosis, it is unclear why this trend should apply to the Danish context alone. Numerous informal barriers to care that prevent or delay healthcare usage have been identified for minority groups [43], and adverse pathways involving police involvement or involuntary admissions, and which do not involve contacts with general practitioners, are more common in some minority groups [44, 45], including refugees and migrants in Denmark and Sweden [46, 47]. Even in a context of full entitlement to healthcare, limited familiarity with or trust towards the mental healthcare system among immediate family members may impair help-seeking, as significant others tend to play a crucial role in initiating and persisting in what can be experienced as a complex process of help-seeking [48, 49]. Minority individuals may also be more likely to feel misunderstood or unwelcome in mental healthcare settings, and such experiences can inform future expectations and help-seeking behaviour [50].

It is unlikely that the higher rates of NAPD contacts in Denmark can be explained by a greater burden of disease alone, nor that the age of onset differs greatly between the two countries. In fact, the one-year prevalence among 20 and 50-year-olds (Figures A1 and A2), respectively, showed that the higher rates in Denmark were specific for youth. Over the full life span, incidence may therefore be similar or even lower in Denmark. In both Denmark and Sweden, validation studies suggest that register-based schizophrenia and schizophrenia-spectrum diagnoses have high reliability in terms of positive predicted values [51–55], but specificity appears lower [56] and is essentially unknown as it depends on whether and when individuals present for treatment. This suggests that under-estimation of true incidence is a greater concern than over-estimation in both countries.

The study therefore underscores the importance of the healthcare context in the interpretation of rates estimated with register data. There is a substantial variation in rates of NAPD reported in the international literature that, in part, has been attributed to differences in study designs [10, 57]. Several direct comparisons of incidence rates suggest a higher average age of onset and two- to three-fold higher estimates in population-based studies using administrative records than in first-contact studies where subjects are screened when they first present for treatment [58, 59]. It a has been suggested that population-based studies capture onset pathways not visible to first-contact studies where clinical criteria at first contact, rather than ultimate diagnosis, determines inclusion [60]. The impact of the study design may even be differential between groups within a population due to differences in care pathways [61]. Adding to these important findings, our study highlights that population-based studies using register data are sensitive to differences in diagnostic practices and group-specific healthcare barriers and contact patterns. Comparative designs may therefore shed light not only on the burden of disease across populations, but also on the role of the healthcare context in rendering mental disorders measurable in administrative records.

### Strengths and limitations

A key strength of this study is that we used high-quality register data harmonized for cross-country comparison. This longitudinal sample included more than four million individuals and twenty thousand cases of NAPD followed for up to 13 years.

A number of limitations deserve mention. First-contacts were defined based on a three-year washout period. This meant that information on psychiatric contact before this three-year period was ignored. A criterion based on first ever registered contact has been used by others [62] and would be more accurate for those segments of the population where longer disease histories were known. However, it would entail uneven washout periods for long-time and more recent residents, as well as for study participants during the end of the study period compared with the beginning [63]. Group definitions were partially based on ground of residence data, but also partly on country of origin, especially in Denmark, which may imply a greater risk of misclassification. Such misclassification could bias downward the difference between the refugee group and the migrant group. For the individual-level adjusted analyses, covariates were measured crudely, wherefore residual confounding for these factors is possible. Although unemployment, sickness-absence and disability pension were measured the year prior to case-ascertainment, it is possible that some individuals already experienced prodromal symptoms at this time leading to reverse causation. The study did not include information on secondary diagnoses or healthcare contacts in the primary sector. However, in both Denmark and Sweden patients suspected of psychotic disorders should be referred to specialized psychiatry. In addition, while general practitioners act as gatekeepers to specialized care in Denmark, they do not as a rule have this role in Sweden, and patients with serious mental health problems would generally bypass primary care and seek care in specialized psychiatry directly [64, 65]. So if anything, primary care data would be expected to increase rather than decrease the difference between the two countries. Another source of information bias inherent to analyses of contact-based data is that individuals with symptoms who have not received treatment are by definition excluded. For other types of disorders, such as affective or stress-related disorders, rates of psychiatric contacts in minority populations do appear underestimated as compared with estimates obtained through primary data collection [66]. However, due to the severity of psychotic disorders we expect this bias to be weaker in studies of NAPD patients. Finally, it is well known that main diagnoses are missing from a substantial portion of outpatient contacts in the Swedish patient registry in the beginning of the study period [67]. Missing diagnoses amounts to as many as 50% of outpatient contacts in 2005, but coverage has gradually improved and in 2013 the proportion of missing was below 10%. This change in coverage partially explains the very large increase in psychiatric contact over time in Sweden, but for NAPD this increase was only observed among 50-year-olds and not among 20-year-olds, which suggests that these diagnoses were not widely used in outpatient care among young patients.

## Conclusion

While corroborating previous findings of increased rates in refugees, our findings also show that the difference in rates between majority and the three non-majority groups were larger than between refugees, non-refuge migrants and descendants of non-refugee migrants. This finding points towards the importance of exposures shared between these three groups that play out within the country of residence, in which all groups spent a portion of their childhood, and can be addressed within that context. Further, the study shows that in Denmark, where early intervention for first-episode psychosis has received increased attention since the late 1990s, rates of NAPD among youth were higher than in Sweden and more concentrated at younger ages. However, this trend was most notable in the majority group. This raises the question whether minorities experience barriers and delays in accessing early treatment. Future studies should investigate how pathways to care and duration of untreated psychosis differ between majority and minority youth, as well as the consequences such inequalities have for both the degree of social marginalization experienced before entering treatment and for life outcomes in the short, medium and long term.

### Supplementary Information

Below is the link to the electronic supplementary material.

Supplementary file1 (DOCX 126 KB)

## Data Availability

Data were obtained through public agencies in Denmark and Sweden and cannot be shared legally. Other researchers can independently apply for access directly from Statistics Denmark and Statistics Sweden if they satisfy the legal criteria for access.
