# Supplementary material for: Incidence of non-affective psychotic disorders in refugees and peers growing up in Denmark and Sweden: a registry linkage study
Source: Soc Psychiatry Psychiatr Epidemiol. 2023 Nov 2;59(7):1153–65. doi: 10.1007/s00127-023-02578-x (PMC11178564; doi:10.1007/s00127-023-02578-x)

**Supplementary material**

**Table A1**. Incidence Rate Ratios (IRR), 95% confidence intervals and p-values. Poisson model offsetting for person-time. Aggregated, pooled data.

**Table A2**. Incidence Rates (IR) and 95% confidence intervals. Linear marginal predictions based on Poisson model offsetting for person-time. Aggregated, pooled data.

**Figure A1**. One year prevalence of any psychiatric and non-affective psychotic (ICD-10 codes F20-F29) contacts among all residents aged 20 in Denmark and Sweden during 2003-2018.

**Figure A2**. One year prevalence of any psychiatric and non-affective psychotic (ICD-10 codes F20-F29) contacts among all residents aged 50 in Denmark and Sweden during 2003-2018.

**Table A1**. Incidence Rate Ratios (IRR), 95% confidence intervals and p-values. Poisson model offsetting for person-time. Aggregated, pooled data.

|  |  | | **Final model** | | |  |  | **- Continued -** | | |
| --- | --- | --- | --- | --- | --- | --- | --- | --- | --- | --- |
|  | | | **IRR** | **p-value** | **[95% CI]** |  | | **IRR** | **p-value** | **[95% CI]** |
| *Group* | | |  |  |  | *Group#sex* | |  |  |  |
|  | *Majority* | | *--- reference ---* | | |  | *Ref#women* | 0.64 | 0.000 | [0.53-0.76] |
|  | *Refugees* | | 1.62 | 0.000 | [1.30-2.00] |  | *Mig#women* | 0.72 | 0.001 | [0.59-0.88] |
|  | *Migrants* | | 1.55 | 0.000 | [1.22-1.97] |  | *Des#women* | 0.64 | 0.000 | [0.51-0.80] |
|  | *Descendants* | | 1.20 | 0.158 | [0.93-1.54] | *Group#country* | |  |  |  |
| *Age* | | |  |  |  |  | *Ref#Sw* | 1.86 | 0.000 | [1.43-2.41] |
|  | *18-20* | | *--- reference ---* | | |  | *Mig#Sw* | 1.33 | 0.061 | [0.99-1.80] |
|  | *21-23* | | 1.18 | 0.000 | [1.10-1.27] |  | *Des#Sw* | 1.23 | 0.429 | [0.74-2.05] |
|  | *24-26* | | 0.93 | 0.060 | [0.86-1.00] | *Age#country* | |  |  |  |
|  | *27-29* | | 0.68 | 0.000 | [0.62-0.73] |  | *21-23#Sw* | 1.08 | 0.194 | [0.96-1.21] |
|  | *30-32* | | 0.53 | 0.000 | [0.48-0.58] |  | *24-26#Sw* | 1.38 | 0.000 | [1.22-1.55] |
|  | *33-35* | | 0.41 | 0.000 | [0.38-0.46] |  | *27-29#Sw* | 1.71 | 0.000 | [1.51-1.94] |
| *Sex* | | |  |  |  |  | *30-32#Sw* | 1.71 | 0.000 | [1.49-1.96] |
|  | *Men* | | *--- reference ---* | | |  | *33-35#Sw* | 1.86 | 0.000 | [1.62-2.15] |
|  | *Women* | | 1.04 | 0.262 | [0.97-1.12] | *Group#age#country* | |  |  |  |
| *Country* | | |  |  |  |  | *Ref#21-23#Sw* | 0.82 | 0.212 | [0.59-1.12] |
|  | *Denmark* | | *--- reference ---* | | |  | *Ref#24-26#Sw* | 0.54 | 0.000 | [0.39-0.74] |
|  | *Sweden* | | 0.34 | 0.000 | [0.31-0.37] |  | *Ref#27-29#Sw* | 0.45 | 0.000 | [0.32-0.63] |
| *Group # age* | | |  |  |  |  | *Ref#30-32#Sw* | 0.44 | 0.000 | [0.30-0.64] |
|  | *Ref#21-23* | | 1.24 | 0.110 | [0.95-1.60] |  | *Ref#33-35#Sw* | 0.31 | 0.000 | [0.20-0.47] |
|  | *Ref#24-26* | | 1.61 | 0.000 | [1.24-2.10] |  | *Mig#21-23#Sw* | 0.89 | 0.551 | [0.62-1.29] |
|  | *Ref#27-29* | | 2.09 | 0.000 | [1.59-2.76] |  | *Mig#24-26#Sw* | 0.80 | 0.259 | [0.55-1.18] |
|  | *Ref#30-32* | | 2.44 | 0.000 | [1.80-3.30] |  | *Mig#27-29#Sw* | 0.38 | 0.000 | [0.26-0.56] |
|  | *Ref#33-35* | | 2.73 | 0.000 | [1.92-3.88] |  | *Mig#30-32#Sw* | 0.36 | 0.000 | [0.24-0.53] |
|  | *Mig#21-23* | | 1.24 | 0.141 | [0.93-1.65] |  | *Mig#33-35#Sw* | 0.49 | 0.004 | [0.30-0.80] |
|  | *Mig#24-26* | | 1.33 | 0.073 | [0.97-1.81] |  | *Des#21-23#Sw* | 1.03 | 0.920 | [0.56-1.91] |
|  | *Mig#27-29* | | 2.02 | 0.000 | [1.48-2.76] |  | *Des#24-26#Sw* | 0.78 | 0.463 | [0.41-1.50] |
|  | *Mig#30-32* | | 2.45 | 0.000 | [1.75-3.42] |  | *Des#27-29#Sw* | 0.74 | 0.401 | [0.37-1.49] |
|  | *Mig#33-35* | | 1.73 | 0.013 | [1.12-2.67] |  | *Des#30-32#Sw* | 0.91 | 0.797 | [0.44-1.88] |
|  | *Des#21-23* | | 1.50 | 0.008 | [1.11-2.03] |  | *Des#33-35#Sw* | 0.52 | 0.097 | [0.24-1.12] |
|  | *Des#24-26* | | 1.72 | 0.001 | [1.24-2.39] | *Group#sex#country* | |  |  |  |
|  | *Des#27-29* | | 1.80 | 0.002 | [1.24-2.62] |  | *Ref#women#Sw* | 1.26 | 0.037 | [1.01-1.58] |
|  | *Des#30-32* | | 2.13 | 0.000 | [1.40-3.23] |  | *Mig#women#Sw* | 1.51 | 0.000 | [1.20-1.89] |
|  | *Des#33-35* | | 3.93 | 0.000 | [2.61-5.91] |  | *Des#women#Sw* | 1.21 | 0.363 | [0.80-1.83] |
| *Age#sex* | | |  |  |  | *Age#sex#country* | |  |  |  |
|  | *21-23#women* | | 0.77 | 0.000 | [0.70-0.85] |  | *21-23#women#Sw* | 1.13 | 0.171 | [0.95-1.34] |
|  | *24-26#women* | | 0.61 | 0.000 | [0.55-0.69] |  | *24-26#women#Sw* | 1.32 | 0.003 | [1.10-1.58] |
|  | *27-29#women* | | 0.60 | 0.000 | [0.53-0.68] |  | *27-29#women#Sw* | 1.53 | 0.000 | [1.27-1.85] |
|  | *30-32#women* | | 0.56 | 0.000 | [0.49-0.65] |  | *30-32#women#Sw* | 1.96 | 0.000 | [1.60-2.39] |
|  | *33-35#women* | | 0.54 | 0.000 | [0.46-0.62] |  | *33-35#women#Sw* | 2.43 | 0.000 | [1.96-3.01] |
| *Sex#country* | | |  |  |  |  |  |  |  |  |
|  | | *Women#Sw* | 0.57 | 0.000 | [0.50-0.65] |  |  |  |  |  |

**Table A2**. Incidence Rates (IRR) and 95% confidence intervals. Linear marginal predictions based on Poisson model offsetting for person-time. Aggregated, pooled data.

|  |  | **MEN** | | | |  | **WOMEN** | | | |
| --- | --- | --- | --- | --- | --- | --- | --- | --- | --- | --- |
|  | **AGE** | **IR ^a^**  Denmark | [95% CI] | **IR** ^a^ Sweden | [95% CI] |  | **IR** ^a^ Denmark | [95% CI] | **IR ^a^**  Sweden | [95% CI] |
| **Majority** | 18-20 | 136 | [129-143] | 46 | [43-49] |  | 142 | [134-149] | 27 | [25-30] |
|  | 21-23 | 160 | [152-168] | 58 | [55-62] |  | 128 | [121-136] | 30 | [28-33] |
|  | 24-26 | 126 | [119-133] | 59 | [55-62] |  | 81 | [75-86] | 28 | [26-31] |
|  | 27-29 | 92 | [86-98] | 53 | [49-56] |  | 57 | [52-62] | 29 | [26-31] |
|  | 30-32 | 72 | [66-77] | 41 | [38-44] |  | 42 | [38-46] | 27 | [25-30] |
|  | 33-35 | 56 | [52-61] | 35 | [33-38] |  | 31 | [28-35] | 27 | [25-30] |
| **Refugees** | 18-20 | 219 | [173-266] | 138 | [119-157] |  | 145 | [112-179] | 66 | [55-77] |
|  | 21-23 | 320 | [267-372] | 177 | [157-197] |  | 163 | [130-196] | 73 | [62-85] |
|  | 24-26 | 329 | [274-384] | 152 | [133-172] |  | 134 | [105-162] | 59 | [49-69] |
|  | 27-29 | 310 | [253-367] | 150 | [129-170] |  | 123 | [95-152] | 66 | [55-77] |
|  | 30-32 | 282 | [220-343] | 133 | [113-170] |  | 105 | [77-133] | 70 | [57-83] |
|  | 33-35 | 211 | [161-261] | 89 | [70-108] |  | 88 | [60-117] | 56 | [43-68] |
| **Migrants** | 18-20 | 211 | [161-261] | 95 | [78-112] |  | 159 | [119-198] | 62 | [50-73] |
|  | 21-23 | 308 | [250-367] | 134 | [114-153] |  | 179 | [140-217] | 75 | [63-88] |
|  | 24-26 | 260 | [204-317] | 129 | [112-147] |  | 120 | [90-150] | 68 | [58-78] |
|  | 27-29 | 287 | [224-351] | 84 | [73-95] |  | 130 | [96-163] | 50 | [43-57] |
|  | 30-32 | 272 | [204-339] | 74 | [65-84] |  | 115 | [82-147] | 53 | [46-60] |
|  | 33-35 | 152 | [95-208] | 63 | [55-71] |  | 61 | [37-85] | 53 | [46-60] |
| **Descendants** | 18-20 | 163 | [122-203] | 68 | [38-97] |  | 109 | [79-138] | 31 | [16-47] |
|  | 21-23 | 288 | [230-346] | 133 | [89-177] |  | 148 | [113-184] | 53 | [32-75] |
|  | 24-26 | 261 | [200-321] | 117 | [74-159] |  | 107 | [77-136] | 44 | [24-63] |
|  | 27-29 | 198 | [140-255] | 104 | [62-146] |  | 79 | [53-106] | 44 | [24-65] |
|  | 30-32 | 182 | [119-245] | 118 | [69-167] |  | 68 | [42-95] | 60 | [32-88] |
|  | 33-35 | 265 | [177-353] | 107 | [54-160] |  | 95 | [59-130] | 64 | [31-98] |
| **Notes:** ^a^ Incidence rate per 100,000 person-years. | | | | | | | | | | |

**Figure A1**. One year prevalence of any psychiatric and non-affective psychotic (ICD-10 codes F20-F29) contacts among all residents aged 20 in Denmark and Sweden during 2003-2018.


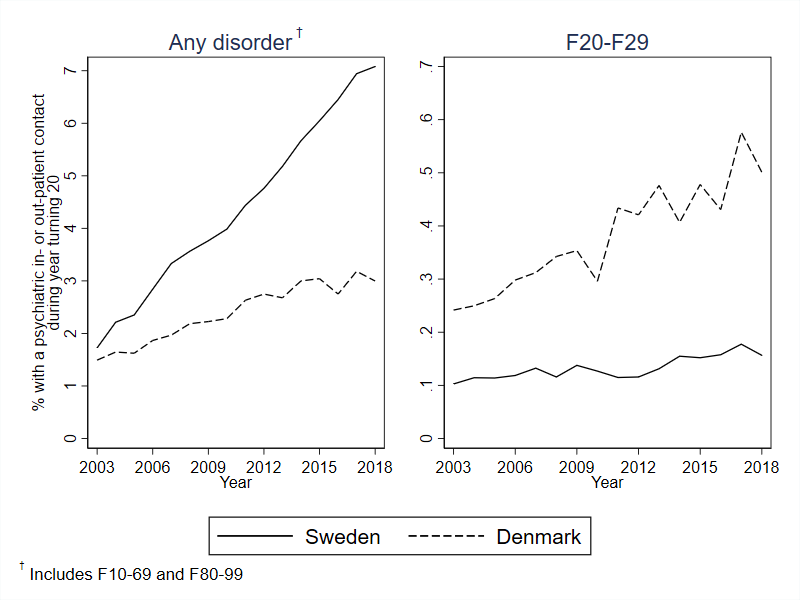


**Figure A2**. One year prevalence of any psychiatric and non-affective psychotic (ICD-10 codes F20-F29) contacts among all residents aged 50 in Denmark and Sweden during 2003-2018.


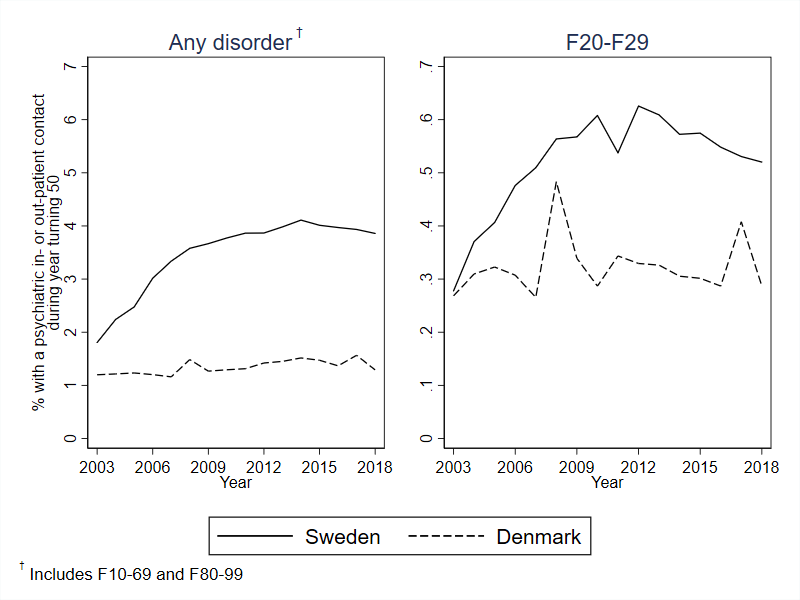

Supplement: Supplementary file 1 — Supplementary file1 (DOCX 126 KB) [file 127_2023_2578_MOESM1_ESM.docx]
